# Stable bloodstream infection rates despite rising colonization: insights from a hospital system using in-house polymerase chain reaction screening for *Candidozyma auris*

**DOI:** 10.1017/ash.2025.10076

**Published:** 2025-08-26

**Authors:** Rossana Rosa, Gemma Rosello, Kelley Manzanillo, Octavio V. Martinez, Lilian M. Abbo

**Affiliations:** 1 Department of Medicine, Horizon Health Network, Fredericton, NB, Canada; 2 Faculty of Medicine, Memorial University of Newfoundland, St John’s, NL, Canada; 3 Department of Medicine, Dalhousie Medicine New Brunswick, Dalhousie University, Halifax, NS, Canada; 4 Infection Prevention and Control Program, Jackson Health System, Miami, FL, USA; 5 Microbiology section, Jackson Memorial Hospital, Miami, FL, USA; 6 Department of Pathology and Laboratory Medicine, University of Miami Miller School of Medicine, Miami, FL, USA; 7 Division of Infectious Diseases, Department of Medicine, University of Miami Miller School of Medicine, Miami, FL, USA

## Abstract

Following the implementation of in-house *Candidozyma auris* (*C. auris*) polymerase chain reaction as part of our surveillance strategy, we observed increasing incidence rates of colonization with *C. auris* already present at hospital admission, while rates of hospital-onset clinical cultures remained stable. Timely detection of *C. auris* colonization can potentially mitigate horizontal transmission.

## Introduction

*Candidozyma auris* (*C. auris*) is spreading in the United States at a rapid pace.^1,2^ Risk-based admission screening for colonization with *C. auris* has been utilized by many institutions to rapidly identify patients colonized with this organism and to efficiently deploy infection prevention resources.^3–5^ We previously described the validation and implementation of a laboratory-developed in-house polymerase chain reaction (PCR) for *C. auris* screening.^4^ This effort was pursued due to lengthy turnaround times when sending out samples to a reference laboratory. Immediately after the implementation of in-house testing, we saw an increase in the rates of *C. auris* colonization already present on admission (POA). We now seek to expand on our experience with this surveillance strategy by describing the observed trends in the rates of newly detected cases of *C. auris* colonization POA, as well as hospital-onset clinical cultures at our facilities since implementing in-house PCR testing.

## Methods

Cross-sectional study conducted at an integrated health system in Miami, Florida, USA, including a large county hospital with a trauma center, burn unit, and transplant service, 3 community hospitals, and an inpatient rehabilitation hospital, totaling approximately 2,500 beds. The study encompassed the period from August 1, 2021, to December 31, 2023.

At our facilities, screening for *C. auris* is performed using a 2-step process previously described and summarized in Supplementary Figure 1.^4^ Briefly, upon admission, all patients undergo a questionnaire embedded in the electronic medical record (EMR) and administered by nursing staff. Patients with at least one risk factor for *C. auris* undergo PCR testing with an axilla-groin swab. As stated, this PCR assay was laboratory-developed, using reagents by DiaSorin (DiaSorin, Cypress, CA), and loaded on the LIAISON MDX thermocycler (DiaSorin, Cypress, CA). The PCR test is processed at the central microbiology laboratory for the health system once a day. Contact isolation is used while awaiting results and continued for the duration of hospitalization in those PCR-positive. An alert is added to the EMR for patients with *C. auris* carriage, and in case of discharge and readmission, patients are re-isolated. At our facilities, all hospitalized patients are bathed daily with chlorhexidine gluconate as part of routine care, but no *C. auris*-specific decolonization strategies are pursued.

**Figure 1.**
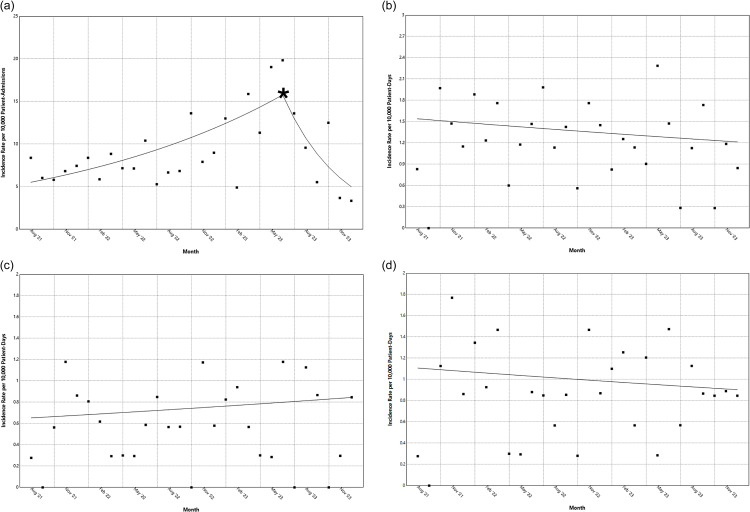
*Candidozyma auris* (*C. auris*) incidence rates of: (***a***) Colonization newly detected as present on admission. (***b***) Hospital-onset clinical cultures. (***c***) Hospital-acquired bloodstream infections. (***d***) Hospital-onset bloodstream infections. Jackson Health System, August 2021 to December 2023. The solid line represents the trend identified by the joinpoint analysis. The * indicates a significant change in trend.

Clinical specimens are processed at the central microbiology laboratory using conventional methods, and isolate identification is performed by matrix-assisted laser desorption ionization–time-of-flight mass spectrometry (MALDI-TOF MS, Biomerieux, Marcy-L’Etoil, France).

The definitions used for the study are presented in Table 1. The outcomes evaluated were rates of *C. auris* POA per 10,000 patient-admissions, rates of *C. auris* hospital-onset culture (HO-C) per 10,000 patient-days, *C. auris* hospital-acquired bloodstream infection (HA-BSI) per 10,000 patient-days, and *C. auris* hospital-onset bloodstream infection (HO-BSI) per 10,000 patient-days.

**Table 1.** *Candidozyma auris* (*C. auris*) outcome definitions

Outcome	Definition
*C. auris* present on admission:	*C. auris* detected on admission screening by polymerase chain reaction in a patient not previously known to be colonized or infected with *C. auris*. Only the first positive test per patient was included.
*C. auris* hospital-onset culture:	*C. auris* identified from a clinical culture collected on or after day 3 of hospitalization in a patient not previously known to be infected or colonized with *C. auris*. Only the first positive culture per patient was included.
*C. auris* hospital-acquired bloodstream infection:	*C. auris* identified from a blood culture collected on or after day 3 of hospitalization in a patient not previously known to be infected or colonized with *C. auris*. Only the first positive culture per patient was included.
*C. auris* hospital-onset bloodstream infection:	*C. auris* identified from a blood culture collected on or after day 3 of hospitalization regardless of a patient’s colonization or infection status with *C. auris*.

We analyzed the monthly trends in the rates of the outcomes of interest with joinpoint regression, a statistical method that allows for breaking the data into time segments to identify points with a statistically significant change in trend. We used the Joinpoint Regression Program (version 5.2, National Cancer Institute, Bethesda, Maryland, USA). This study received approval from the Institutional Review Board.

## Results

We identified 292 unique patients with *C. auris* POA who were not previously known to be carriers, for an overall incidence rate of 9.1 per 10,000 patient-admissions. Joinpoint regression analysis showed that between August 2021 and May 2023, there was a monthly percent increase in rates of 4.9% (CI 3.0–8.8; *p* < .001), followed by a decrease in rates of 17.4% (CI −52.4–−5.6; *p* = .01) from July 2023 to December 2023 (Figure 1A). Notably, CA-POA incidence rates during May and June 2023 were 19.04 and 19.85 per 10,000 patient-admissions, respectively, which were the highest observed throughout the study period, and the rates observed in subsequent months were within the range of the observed rates in the preceding months.

We observed 122 *C. auris* HO-C, for an overall incidence rate of 1.2 per 10,000 patient-days, and no changes in trend were identified (monthly percent change −.8 [CI -2.9–1.1;*p* = .36]) (Figure 1B). There were 58 *C. auris* HA-BSI for an incidence rate of .6 per 10,000 patient-days, and no changes in trend were identified (monthly percent change .9 [CI -1.1–3.2; *p* = .36]) (Figure 1C). A total of 87 *C. auris* HO-BSI were identified for an incidence rate of .9 per 10,000 patient-days, and no changes in trend were detected during the study period (monthly percent change −.72 [CI −3.1–1.7; *p* = .50) (Figure 1D).

## Discussion

*C. auris* continues to rapidly spread throughout our community as evidenced by the rising rates of newly detected patients colonized with this organism. Most cases seen in our region are related to transmission in healthcare settings, particularly nursing homes and ventilator-capable facilities.^6^ A US nationwide wastewater monitoring study conducted between 2023 and 2024 showed a higher frequency of *C. auris* detection in sewersheds with a higher number of nursing homes and hospitals.^2^ The authors also noted a lack of association between the number of wastewater positive samples and the number of case rates as reported in the National Notifiable Disease Surveillance System, likely reflecting limited *C. auris* testing capacity for most hospitals in the United States.^2^ As such, our data, along with other studies of hospital-based *C. auris* surveillance, shows the burden of colonization that healthcare facilities can uncover when proactively testing for *C. auris*.^3–5^

Despite the rising rates of colonization and the observed expansion in the sources of clinical cultures with *C. auris*,^7^ the rates of hospital-onset clinical cultures remained unchanged during the observation period. We hypothesize this could be in part due to in-house testing capabilities for both surveillance PCR and clinical cultures, which facilitate the prompt detection of *C. auris* colonization and the efficient deployment of infection prevention resources, as well as during outbreak response.^4,8^ However, completely eradicating *C. auris* from the hospital environment is very challenging, as illustrated by Sansom *et al.*, who showed that in the rooms of hospitalized patients colonized with *C. auris*, up to 20% of the environmental surfaces were culture-positive 4 hours after disinfection.^9^

Our study has several limitations. First, we present observational data on the temporal trajectory of rates of colonization newly detected on admission and of HO-Cs but do not seek to establish a causal association between a specific screening strategy and clinical outcomes. Also, we did not assess the potential burden from *C. auris* acquisition in the form of skin colonization since we do not re-test patients after the initial screening. Yet, we analyzed rates of bloodstream infections, arguably the most relevant of clinical outcomes arising from acquisition, and found these remained unchanged throughout the observation period. Both clinical cultures and bloodstream infections with *C. auris* remain a rare outcome, which makes it difficult to detect a statistically significant change. Lastly, we report on a single health system experience; nevertheless, our facilities are the safety net system for a large and diverse population.

## Conclusion

Throughout most of the study period, we observed rising rates of patients with *C. auris* newly detected during admission screening, while rates of *C. auris* HO-Cs and bloodstream infections remained stable. Our findings point to the ongoing pressure exerted by the spread of *C. auris* throughout the continuum of healthcare facilities and the efforts required by infection prevention programs to contain it. Further studies are needed to quantify the impact of different screening strategies on *C. auris* acquisition.

## Supporting information

10.1017/ash.2025.10076.sm001 Rosa et al. supplementary material Rosa et al. supplementary material

## Supplementary material

The supplementary material for this article can be found at https://doi.org/10.1017/ash.2025.10076.
