# Supplementary figures and images for: Stable bloodstream infection rates despite rising colonization: insights from a hospital system using in-house polymerase chain reaction screening for Candidozyma auris
Source: Antimicrob Steward Healthc Epidemiol. 2025 Aug 26;5(1):e189. doi: 10.1017/ash.2025.10076 (PMC12394015; doi:10.1017/ash.2025.10076)

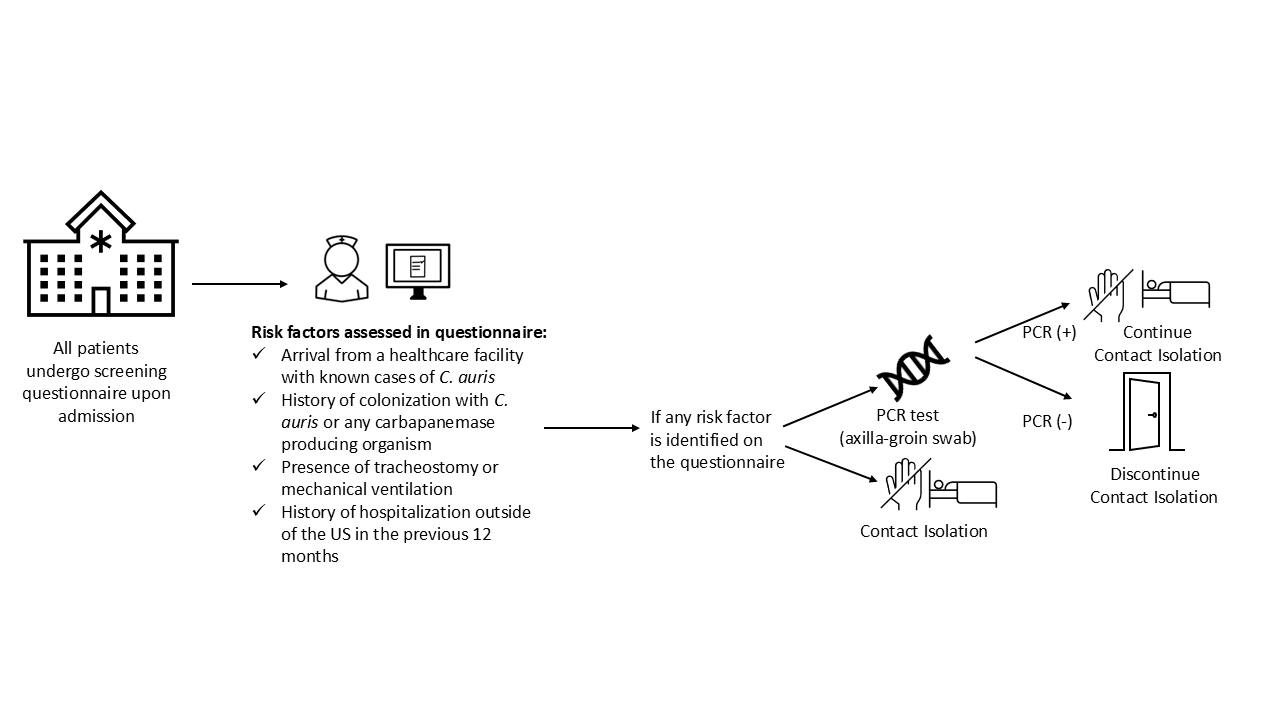

Supplement: Rosa et al. supplementary material [file S2732494X25100764sup001.tif]
